# RETRACTION: Effect of Different Catheter Pathways on Wounds After Minimally Invasive Radical Prostatectomy: A Meta‐Analysis

**DOI:** 10.1111/iwj.70349

**Published:** 2025-03-17

## Abstract

**RETRACTION:** WangT. , JiS. , ZhangC. , XiangY. , and YinG. , “Effect of Different Catheter Pathways on Wounds After Minimally Invasive Radical Prostatectomy: A Meta‐Analysis,” International Wound Journal 21, no. 2 (2024): e14443, 10.1111/iwj.14443.PMC1082812437905390

The above article, published online on 31 October 2023, in Wiley Online Library (http://onlinelibrary.wiley.com/), has been retracted by agreement between the journal Editor in Chief, Professor Keith Harding; and John Wiley & Sons Ltd. Following an investigation by the publisher, all parties have concluded that this article was accepted solely on the basis of a compromised peer review process. The editors have therefore decided to retract the article. The authors did not respond to our notice regarding the retraction.

**RETRACTION:** T. Wang , S. Ji , C. Zhang , Y. Xiang , and G. Yin , “Effect of Different Catheter Pathways on Wounds After Minimally Invasive Radical Prostatectomy: A Meta‐Analysis,” International Wound Journal 21, no. 2 (2024): e14443, 10.1111/iwj.14443.PMC1082812437905390

The above article, published online on 31 October 2023, in Wiley Online Library (http://onlinelibrary.wiley.com/), has been retracted by agreement between the journal Editor in Chief, Professor Keith Harding; and John Wiley & Sons Ltd. Following an investigation by the publisher, all parties have concluded that this article was accepted solely on the basis of a compromised peer review process. The editors have therefore decided to retract the article. The authors did not respond to our notice regarding the retraction.

